# Female gametophyte development in *Sedum sediforme* (Jacq.) Pau (Crassulaceae): an anatomical, cytochemical and ultrastructural analysis

**DOI:** 10.1007/s00709-018-1319-9

**Published:** 2018-10-15

**Authors:** Emilia Brzezicka, Małgorzata Kozieradzka-Kiszkurno

**Affiliations:** Department of Plant Cytology and Embryology, Faculty of Biology, University of Gdańsk, 59 Wita Stwosza St., 80-308 Gdańsk, Poland

**Keywords:** Antipodal cells, Embryo sac, Megagametogenesis, Megasporogenesis, Plasmodesmata, Wall ingrowths

## Abstract

Available documentation about the development of the female gametophyte of Crassulaceae is very limited. The aim of this study was to extend the embryological knowledge of Crassulaceae by analysing the development of the embryo sac in *Sedum sediforme*. Transmission electron microscopy and light microscopy including Nomarski optics (DIC) were used to observe individual stages of female gametophyte development. Cytochemical staining enabled detection of lipids, insoluble polysaccharides and proteins in gametophyte cells during their formation. Their increased accumulation was observed during nucellar cell and unfunctional cell degeneration in the embryo sac at the coenocytic and cellular stages (megagametogenesis). The female gametophyte develops in anatropous, bitegmic and crassinucellate ovules. The mature embryo sac is built of seven cells but after antipodes degeneration it is formed by the egg apparatus and a central cell. The monosporic *Polygonum* type was observed. One megaspore mother cell (MMC) formed three cells after meiosis. A triad was formed from a functional megaspore (placed chalazally), one uninucleate megaspore and a binucleate cell located at the micropylar end. Plasmodesmata with adhering electron-dense dome were noticed in walls of the coenocytic embryo sac and in the outer walls of ephemeral antipodes. Moreover, similar to synergids, antipodes form wall ingrowths. Here, we report new structural features of the antipodal cells (the presence of plasmodesmata with an electron-dense dome) which have not been described before. This new structural observation indicates that these cells participate in substance transport and that this process can probably be additionally regulated.

## Introduction

The haploid gametophyte is one of two generations described during the seed plants life cycle, whose major function and result is the production of female and male gametes (Yadegari and Drews 2004). Gametophyte and consequently gamete production is associated with two processes: sporogenesis and gametogenesis. During sporogenesis, haploid spores are formed after meiosis. Next, the spores develop into gametophytes during gametogenesis. The gametophytes harbour the gametes as an egg cell and a central cell (female gametophyte) and sperm cells (male gametophyte) (Sánchez-León and Vielle-Calzada 2010; Schmidt et al. 2015). In angiosperms, the phenomenon of double fertilisation can be observed, in which one of the sperm cells fuses with an egg cell and a second male gamete fuses with a central cell. Plasma membrane and nuclear fusion are two successively observed processes (Jensen 1973; Raghavan 2000, 2006). Double fertilisation provides the generation of the embryo and endosperm. Plant reproduction is closely related with the female gametophyte. In angiosperms, the female gametophyte is involved in pollen tube reception, control of seed development initiation and both sexual and asexual seed formation (Wang et al. 2006; Drews and Koltunow 2011; Wang et al. 2017). The female gametophyte allows the formation of seeds in which embryos develop either after fertilisation (zygotic embryos) or without fertilisation (during gametophytic apomixis) (Drews and Koltunow 2011; Schmidt et al. 2015).

The female gametophyte (i.e. embryo sac or megagametophyte) (Reiser and Fischer 1993; González-Gutiérrez et al. 2014; Rojek et al. 2018) develops in the nucellus, which is covered with integument(s). The *Polygonum* type of development occurs in most flowering plants, including species of the family Crassulaceae and genus *Sedum* (Mauritzon 1933; Thiede and Eggli 2007). However, megaspores/cells formed during megasporogenesis exhibit variation in number and arrangement (Maheshwari 1937; Bouman 1984). In the monosporic *Polygonum* type, the most frequent arrangement is a linear tetrad of megaspores. In representatives of Crassulaceae, linear, T-shaped, oblique T-shaped and isobilateral (this is doubtful) tetrads have been noticed (Johri et al. 1992). Linear triads have also been observed, where the micropylar cell is mononuclear or binucleated or even with a binucleated middle. One or a maximum of three arrangement types of cells formed after megasporogenesis occur in representatives of the family. In some Crassulaceae species, for example in *Aeonium guttatum*, the formation of female gametophyte from one-nucleate, three-nucleate or four-nucleate cells has been observed during megagametogenesis (Mauritzon 1933).

The monosporic *Polygonum* and bisporic *Allium* type (two species) of female gametophyte development has been observed only in the most species-rich genus—*Sedum* (ca. 420 species)—within Crassulaceae. This is the biggest genus of the 33–35 described in the family (Hart and Bleij 2003; Christenhusz and Byng 2016). Regardless of the type of development (*Polygonum* or *Allium*) the embryo sac is built of seven cells: an egg cell, two synergids, a central cell and three antipodal cells. So far, embryological data, including the ultrastructural and cytochemical information collected for the Crassulaceae family, mainly presents the process of embryogenesis (Kozieradzka-Kiszkurno and Bohdanowicz 2006; Kozieradzka-Kiszkurno et al. 2012; Czaplejewicz and Kozieradzka-Kiszkurno 2013). In contrast, informations about previous processes that occur in the ovule before fertilisation are very limited. The development of the female gametophyte of a few species from Crassulaceae and genus *Sedum* has been subject of research, but this process has been observed only with the use of light microscopy. The available documentation is primarily based on schematic drawings (Sharp 1913; Souéges 1927; Mauritzon 1933; Johri et al. 1992; Wojciechowicz and Samardakiewicz 1998; Thiede and Eggli 2007). The ultrastucture and cytochemistry of Crassulaceae has only been studied with reference to *Sedum hispanicum* L. (Brzezicka and Kozieradzka-Kiszkurno 2018). The paper was the first to report the occurrence of a new kind of plasmodesmata (simple plasmodesmata with an electron-dense dome) during the development of the embryo sac. Compound and simple plasmodesmata with adjacent electron-dense material have been previously described during embryogenesis only in cell walls of suspensors in Crassulaceae (Kozieradzka-Kiszkurno and Bohdanowicz 2010; Kozieradzka-Kiszkurno et al. 2011; Kozieradzka-Kiszkurno and Płachno 2012). The unique structural features observed during embryological studies on Crassulaceae mean that this family is an interesting research area, which would allow a better understanding of the potential function of female gametophyte cells.

*Sedum sediforme* (Jacq.) Pau belongs to the highly polyphyletic genus *Sedum* series *Rupestria* (Nikulin et al. 2016) and family Crassulaceae which comprises approximate 1400 species of dicotyledonous plants (Christenhusz and Byng 2016). *Sedum* series *Rupestria* is a group which is monophyletic and endemic to the Euro-Mediterranean region (Gallo and Zika 2014). So far, studies on *S. sediforme* ovules have been performed during embryogenesis to identify the structure and function of suspensors (Majcher 2017). During embryogenesis, ultrastructural and cytochemical studies have also been conducted on ovules of other species from the series *Rupestria-S. rupestre* L. (Czaplejewicz and Kozieradzka-Kiszkurno 2013). In *S*. *sediforme* and *S. rupestre*, plasmodesmata with an electron-dense material have not been recorded in the cell walls of the suspensor basal cell (Czaplejewicz and Kozieradzka-Kiszkurno 2013; Majcher 2017). So far, a lack of these plasmodesmata has only been observed in representatives of the *Sedum* series *Rupestria* in Crassulaceae and this has meant that this species has become an interesting research topic for us. The different structural features observed in the above-mentioned species during embryogenesis in comparison to the other Crassulaceae may also suggest that at the earlier stages of development (megasporogenesis and megagametogenesis) some new structural features can appear. During our research, we tested whether the absence of plasmodesmata with an electron-dense dome during embryogenesis means that they are missing during female gametophyte development.

In this article, we present an anatomical, cytochemical and ultrastructural study of the female gametophyte in *Sedum* (Crassulaceae). The main purpose of the study was to determine the type of female gametophyte development in *Sedum sediforme* (Jacq.) Pau and gain an understanding of the reproductive processes in Crassulaceae. These studies are the first to be in representatives of *Sedum* series *Rupestria* (*S. sediforme*) during megasporogenesis and megagametogenesis. During the research, we tested the hypothesis that species from genus *Sedum* show variability in the development of the embryo sac. This was tested with regard to the type/manner of female gametophyte development and its structure. The present research is aimed at conducting a first comparative ultrastructural and cytochemical analysis of the female gametophyte within *Sedum*, which will contribute to the determination of structural differences, similarities, and characteristics features for the study species. The observations made during this study are discussed in comparison to embryological data obtained for other angiosperms.

## Materials and methods

### Plant material

To study the development of the female gametophyte in *S. sediforme*, the flowers were harvested both before and at the stage of anthesis. Ovules at different developmental stages were isolated using preparative needles during growing seasons in years 2015–2017. Plants of selected species were obtained from collection of the Botanical Garden of the Jagiellonian University in Kraków (Poland).

### Clearing technique

Developing *S. sediforme* ovules were fixed in methanol:acetic acid (3:1, *v*/*v*) for 24 h and then were cleared as described by Rojek et al. (2018). The material was rinsed successively in 96% (10 min) and 100% (3 times for 10 min) methanol. Then, the specimens were treated with acidulated DMP (dimethyl pimelimidate) overnight and placed in DMP:propylene oxide (1:1, *v*/*v* for 20 min; 1:3 for 20 min) and finally in clear propylene oxide (20 min). The clearing process was carried out gradually in a mixture of cedar oil and propylene oxide (1:10, *v*/*v*) overnight in an open Eppendorf tube. The analysed material was placed in a drop of pure cedar oil on a microscope slide. A light microscope (Nikon Eclipse E 800) with differential interference contrast (DIC) optics was used for observations.

### Electron and light microscopy observations

Procedure used for transmission electron microscopic observations (TEM) and light microscopic analyses (LM) were the same as those used for the study of ovules (during female gametophyte development and embryogenesis (Czaplejewicz and Kozieradzka-Kiszkurno 2013; Brzezicka and Kozieradzka-Kiszkurno 2018) in another species from genus *Sedum*. The first samples were fixed in a mixture of 2.5% glutaraldehyde, 2.5% formaldehyde (prepared from paraformaldehyde) in 0.05 M cacodylate buffer (pH = 7.0) and then rinsed with the same buffer and post-fixed overnight in 1% osmium tetraoxide in cacodylate buffer at 4 °C. Ovules were treated for 1 h with aqueous solution of 1% uranyl acetate (only for TEM) and next dehydrated in an acetone series.

For TEM, specimens were embedded in Spurr’s epoxy resin (Spurr 1969) and cut for ultrathin sections (50–100 nm) with a diamond knife on a Leica EM UC7 ultramicrotome and then post-stained (uranyl acetate in 50% ethanol and 0.04% lead citrate). The sections were examined using a Philips CM 100 and FEI Tecnai G^2^ Spirit TWIN/BioTWIN transmission electron microscope (Faculty of Biology, University of Gdańsk) at an accelerating voltage of 120 kV.

For LM, ovules were also embedded in Spurr’s resin and then were cut into semi-thin sections (0.5–1.5 μm) with a glass knife on a Sorvall MT 2B ultramicrotome. Sections were stained using toluidine blue O (TBO) for control, periodic acid-Schiff (PAS) reagent (Jensen 1962) for water-insoluble polysaccharides detection, aniline blue black (ABB; Jensen 1962) for proteins identification, and with Sudan black B (SBB; Bronner 1975) for checking for the presence of lipids. Sections were analysed and photographed with a Nikon Eclipse E 800 light microscope equipped with a Nikon DS-5Mc camera using Lucia Image software.

## Results

### Megasporogenesis and selection of the functional megaspore

**Fig. 1:**
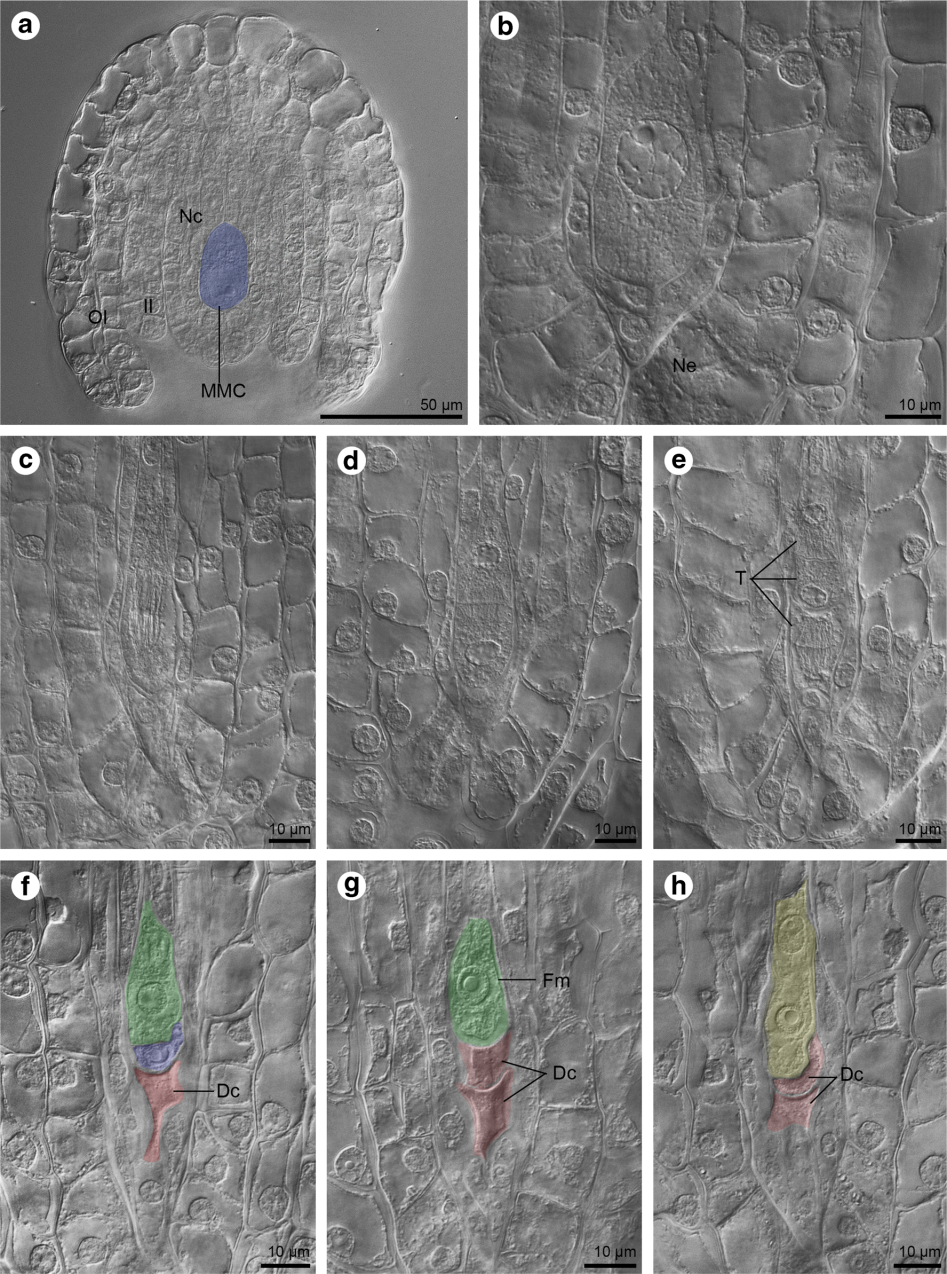
Ovules of *Sedum sediforme* (Jacq.) Pau cleared during the formation of a functional megaspore and two-nucleate gametophyte. **a**–**h** DIC-image. **a** Cleared ovule showing the megaspore mother cell (MMC, blue). The nucellar cells (Nc), inner (II) and outer (OI) integuments are visible. **b** The megaspore mother cell located within the nucellus; (Ne) nucellar epidermis. **c** The megaspore mother cell during division—elongated meiocyte. **d** Result of first meiotic division—dyad stage. **e** Triad (T) formation. **f** Triad built of the functional megaspore located chalazally (green), uninucleate megaspore placed in the middle of the triad (blue) and binucleate cell at the micropylar region (red). The micropylar one starts the process of degeneration (Dc). **g** Functional megaspore (green, Fm) localised chalazally and two degenerating cells placed at the micropylar pole (red, Dc). **h** Two-nucleate gametophyte (yellow) formed after mitotic division. Two degenerating cells are located chalazally (red, Dc)

The *Sedum sediforme* female gametophyte develops within the ovule placed in the ovary of the pistil. Ovules of the tested species are bitegmic. Each integument is formed by two layers of cells. The nucellus is successively covered by developing inner and outer integuments during ovule growth (Fig. 1a). In the initial stage of development, the ovule of *S. sediforme* is relatively small. During megasporogenesis, the one megaspore mother cell (MMC) is visible within the nucellar tissue (Fig. 1a–b). It is located below the nucellar epidermis, from which it is separated by one or two layers of cells (Fig. 1a–b). The MMC, also termed the precursor of the meiotic products, is significantly different from the surrounding cells. Compared to neighbouring cells, it is larger and has a clearly marked nucleus (Fig. 1b). During development, the MMC increases in size and elongates in the micropylar-chalazal direction. The division of the nucleus is seen approximately in the central part of the meiocyte during the first meiotic division (Fig. 1c). Two mononuclear cells are formed after karyokinesis, followed by cytokinesis (dyad) (Fig. 1d). Next, the chalazal cell divides and forms two mononuclear megaspores. The nucleus of the micropylar cell divides; however, a phragmoplast structure is not present in the binucleate micropylar cell and a cell plate is not formed (Fig. 1e). So, the result of the second meiotic division is a linear triad of cells (two megaspores and a binucleate micropylar cell) which are arranged in accordance with the micropylar-chalazal axis (Fig. 1e). The embryo sac mother cell that initiates the megagametogenesis is one of the cells of the triad (Fig. 1f–g). The functional megaspore placed on the chalazal side is always accompanied by two degenerating cells on the micropylar side. The time of their degeneration can be different. The micropylar cell degenerates first (Fig. 1f). The *S. sediforme* embryo sac develops from a one-nucleate functional megaspore which is localised chalazally—monosporic megasporogenesis (Fig. 1g). A binucleate embryo sac is formed by the first mitotic division of the embryo sac mother cell (Fig. 1h).

### Megasporogenesis—cytochemical and ultrastructural analysis

**Fig. 2:**
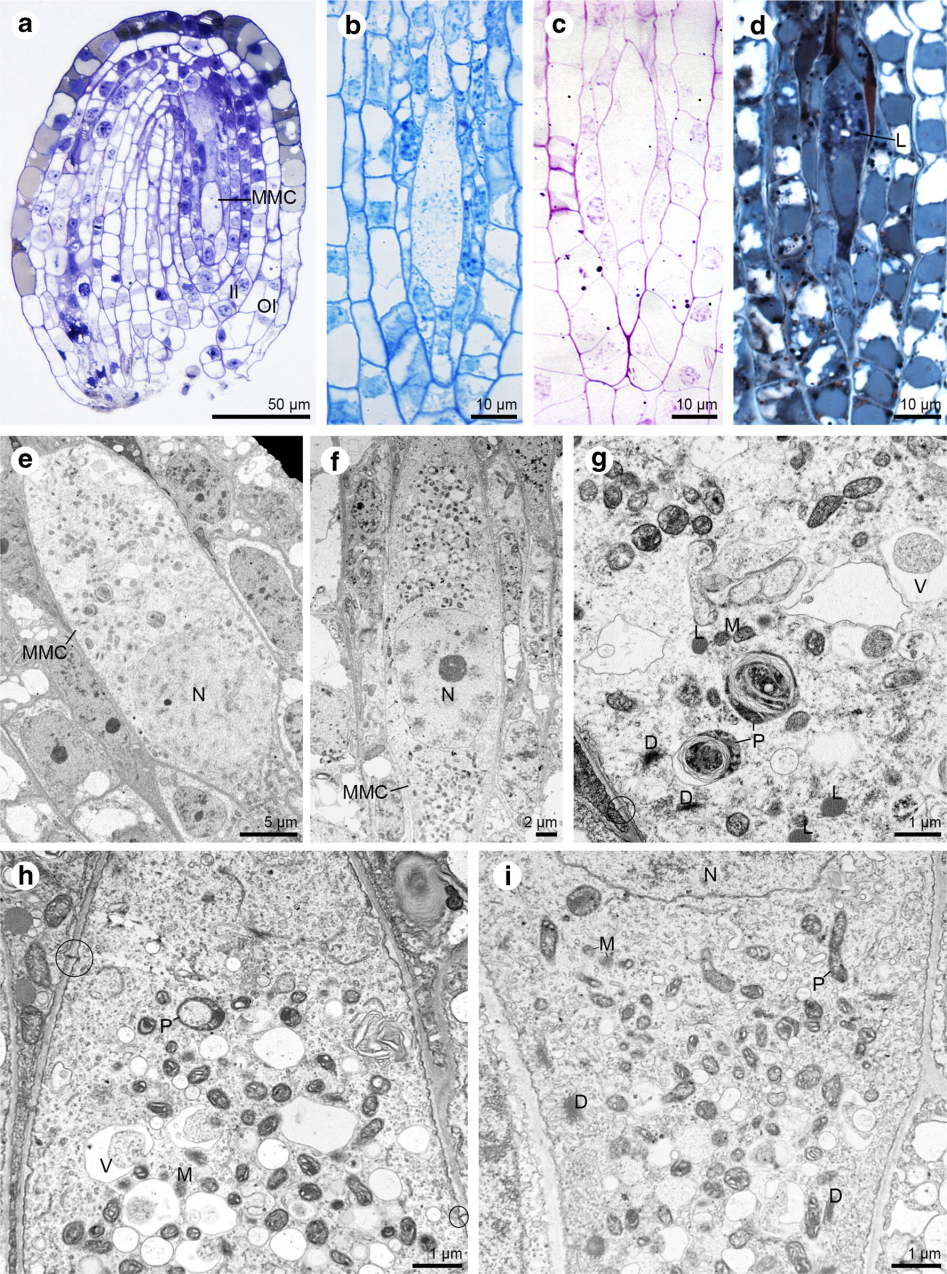
Ovule of *Sedum sediforme* (Jacq.) Pau at the megaspore mother cell stage. **a**–**d** Light micrographs—results of cytochemical tests; **e**–**i** Electron micrographs. **a** Longitudinal section of anatropous, crassinucellate and bitegmic ovule at the megaspore mother cell (MMC) stage. The outer (OI) and inner (II) integuments are observed. **b** Protein detection in megaspore mother cell after aniline blue black (ABB) staining. **c** Section stained with the PAS method which shows the presence of starch grains within megaspore mother cell cytoplasm. **d** Lipid droplets (L) detection in megaspore mother cell after Sudan black B staining. **e** Megaspore mother cell (MMC) is visibly enlarged compared to surrounding nucellar cell. Big nucleus (N) is placed in the micropylar region of the cell. **f** Elongated megaspore mother cell (MMC) with centrally located nucleus (N). **g** Magnification of megaspore mother cell form (**e**) with observed plastids (P), lipid droplets (L), dictyosomes (D), mitochondria (M), and vacuoles (V) in the cytoplasm. Plasmodesmata occur in the chalazal walls of megasporocyte (circle). **h** Chalazal part of the elongated megaspore mother cell from (f) with found plastids (P), mitochondria (M), vacuoles (V), and plasmodesmata (circle). **i** Magnification of the micropylar part of megasporocyte with present dictyosomes (D), plastids (P), mitochondria (M), and nucleus (N) present in the cytoplasm

The megaspore mother cell, which initiates the process of megasporogenesis, is clearly distinguished from the remaining ovular cells (Fig. 2a). Cytochemical staining conducted at the stage of the meiocyte revealed the presence of proteins (Fig. 2b), insoluble polysaccharides (Fig. 2c) and lipids (Fig. 2d). A significant sized cell nucleus changed its position depending on the developmental phase (Fig. 2e–f). When the megaspore mother cell was already clearly elongated, its nucleus was located in central part before the first meiotic division. Ultrastructural analysis confirmed the results obtained from cytochemical stainings and allowed observation of the listed substances in the form of small starch granules and lipid droplets (Fig. 2g). In addition, a large nucleus, active dictyosomes, plastids, mitochondria and autophagic vacuoles were found in the cytoplasm of the MMC (Fig. 2g–i). Simple plasmodesmata connected the chalazal part of the megasporocyte with nucellar cells (Fig. 2g–h). Some of the plastids were distinguished by the presence of areas where the content was similar to the surrounding cytoplasm (Fig. 2h). Organelles appeared to be evenly distributed in the cytoplasm at both poles of the meiocyte (Fig. 2f, h–i).

**Fig. 3:**
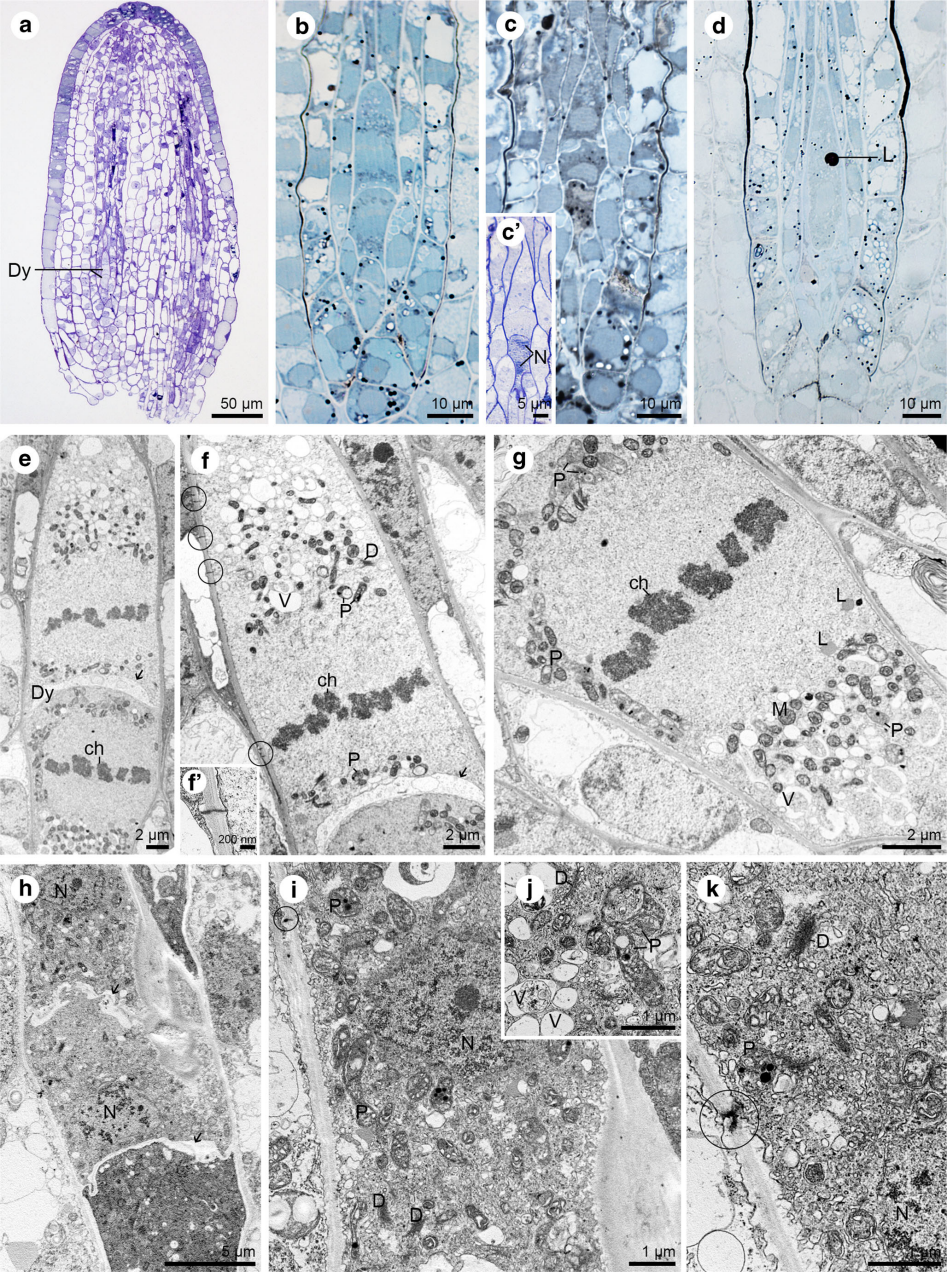
Dyad and triad stage of megasporogenesis in *Sedum sediforme* (Jacq.) Pau. **a**–**d** Light micrographs—results of cytochemical tests; **e**–**k** Electron micrographs. **a** General view of the an anatropous ovule with formed dyad (Dy). **b** Semi-thin section with detected lipids within the dyad cells after Sudan black B staining. **c** Linear triad of cells with lipid droplets present in all formed cells. **c’** Two-nucleate (N) micropylar cell of the triad. **d** Sudan black B staining. The functional megaspore with noted lipid droplets (L) in its cytoplasm. **e** Ultrastructure of dyad (Dy) from (**a** and **b**) with visible thickened cross cell wall (arrow). Both cells of dyad undergo karyokinesis. The condensed chromatin of the chromosomes (ch) is visible. **f** Magnification of chalazal dyad cell with plasmodesmata (circle) located in the cell walls, which separate cell from nucellus. The cross wall of the dyad is thickened (arrow). Cytoplasm contains plastids (P), dictyosomes (D) and many vacuoles (V). The condensed chromatin of the chromosomes (ch) is visible. **f’** Magnification of the plasmodesma from (**f**). **g** Micropylar dyad cell during second meiotic division with observed lipid droplets (L), mitochondria (M), vacuoles (V), and plastids (P) and condensed chromatin of the chromosomes (ch). **h** Section with visible triad. The cells placed chalazally and in the middle of the triad are mononucleate; Nucleus (N), thickened cross wall (arrow). **i** Higher magnification of the chalazal placed megaspore of the tetrad from (**i**). The plastid (P), dictyosomes (D), nucleus (N) and simple plasmodesmata (circle) are visible. **j** Magnification of the cytoplasm content from (i) with plastids (P), active dictyosomes (D) and vacuoles (V) present. **k** Detail of the lateral wall of middle megaspore from (**h**) with noted active dictyosomes (D), plastids (P), nucleus (N) and compound plasmodesmata (circle)

The *S. sediforme* ovule continued to grow during the development of the female gametophyte (Fig. 3a). The implementation of cytochemical staining confirmed that there were also reserved materials in the form of lipid drops at the stage of dyad, triad and functional megaspore (Fig. 3b–d). The cross cell wall formed after first meiotic division and separating cells was clearly thickened by the electron-translucent material (Fig. 3e–f). The nucleus divided in both cells of the dyad (Fig. 3e), but the micropylar cell remained undivided -lack of cytokinesis (Fig. 3c’). Simple plasmodesmata existed in the chalazal wall which separated the cell from circumjacent nucellus (Fig. 3f, f’). In addition, numerous small vacuoles, mitochondria, plastids, lipid droplets, and dictyosomes were observed in both dyad cells (Fig. 3f–g). Also at the triad stage, transverse walls separating the triad cells were clearly thickened and irregularly formed (Fig. 3h). Within the cytoplasm of the chalazal mononuclear triad cell, there were plastids, active dictyosomes, mitochondria (not shown) and vacuoles (Fig. 3i–j). Both at the dyad and triad stage, plastids were recorded with areas similar to the surrounding cytoplasm (Fig. 3f, j). At the triad stage, the plasmodesmata were characterised by a diversified structure. Plasmodesmata observed in the outer walls of the largest, chalazal megaspore were simple (Fig. 3i), while those seen in the outer walls of the middle megaspore of the triad were branched (Fig. 3k).

**Fig. 4:**
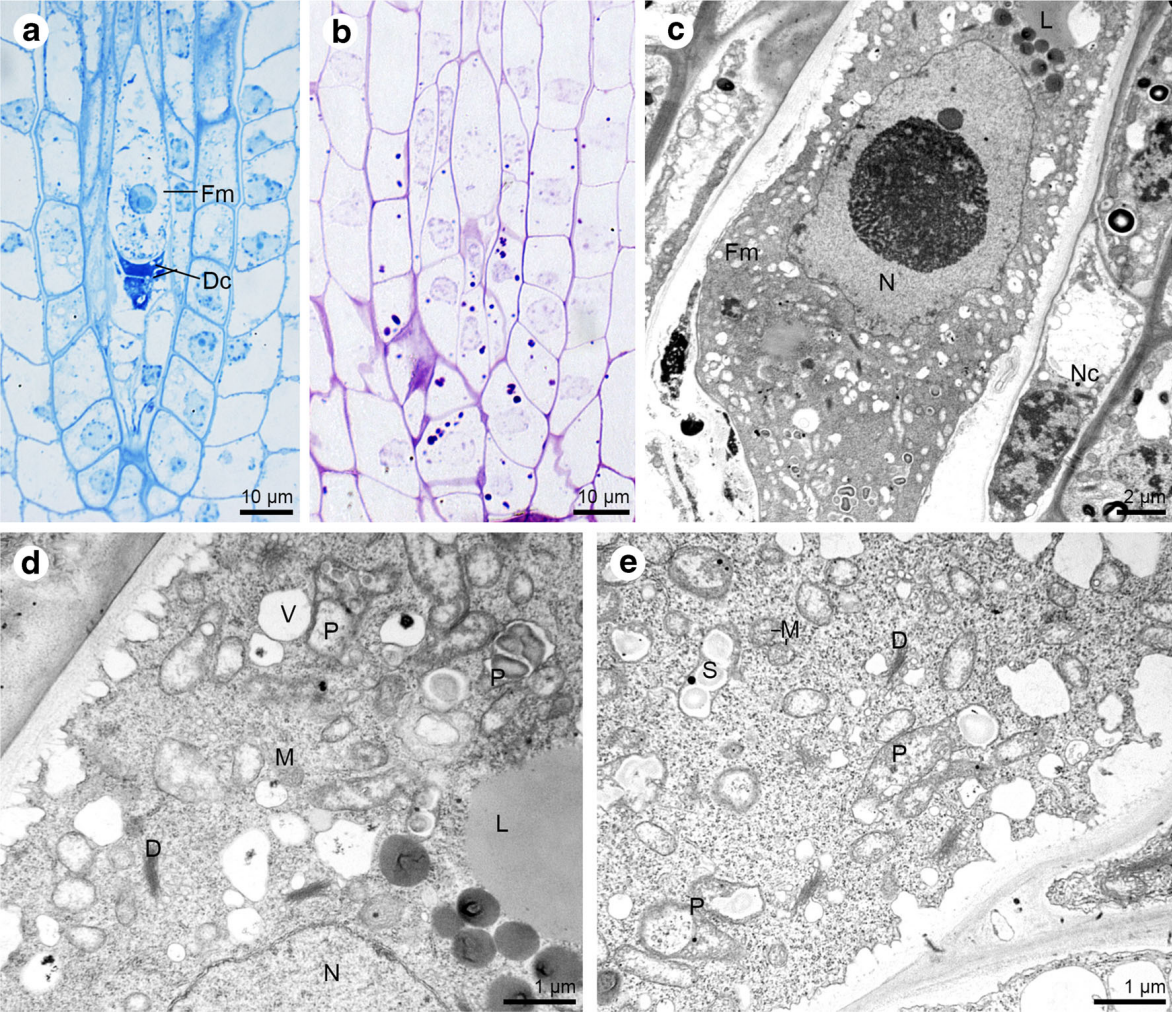
The functional megaspore structure. **a**–**b** Light micrographs—results of cytochemical tests; **c**–**e** Electron micrographs. **a** Two micropylar cells of the triad degenerate (Dc) and stain more intensely for proteins than the functional megaspore (Fm). **b** Functional megaspore staining with periodic acid-Schiff for insoluble polysaccharides. **c** General view of the functional megaspore (Fm) with visible big nucleus (N), lipid droplets (L) and neighbouring nucellar cells (Nc). Some degenerating cells of the triad are observed in the micropylar region. **d** Magnification of the embryo sac mother cell form (**c**). Different sized lipid droplets (L), plastids (P), dictyosomes (D), mitochondria (M) and nucleus (N) can be noticed. **e** Ultrastructure of the functional megaspore cell with plastids (P) with starch grains (S), mitochondria (M), and dictyosomes (D)

The chalazal megaspore provides the origin to the embryo sac mother cell, which is the only megaspore that is not degenerated and undergoes three mitotic divisions (without cytokinesis). It gives a positive reaction after ABB staining and the PAS method, which suggests points on the presence of proteins (Fig. 4a) and insoluble polysaccharides (Fig. 4b). The degenerating cells stain intensively compared to the functional megaspore. The cell walls of the functional megaspore are unevenly thickened (Fig. 4c–d). Moreover, walls are crossed by plasmodesmata (Fig. 4e). At this stage, the nucleus, plastids (also with intraplastidial space or starch grains), dictyosomes, vacuoles, mitochondria and lipid droplets are present in the cytoplasm of the functional megaspore (Fig. 4d–e).

### Megagametogenesis—cytochemical and ultrastructural analysis

**Fig. 5:**
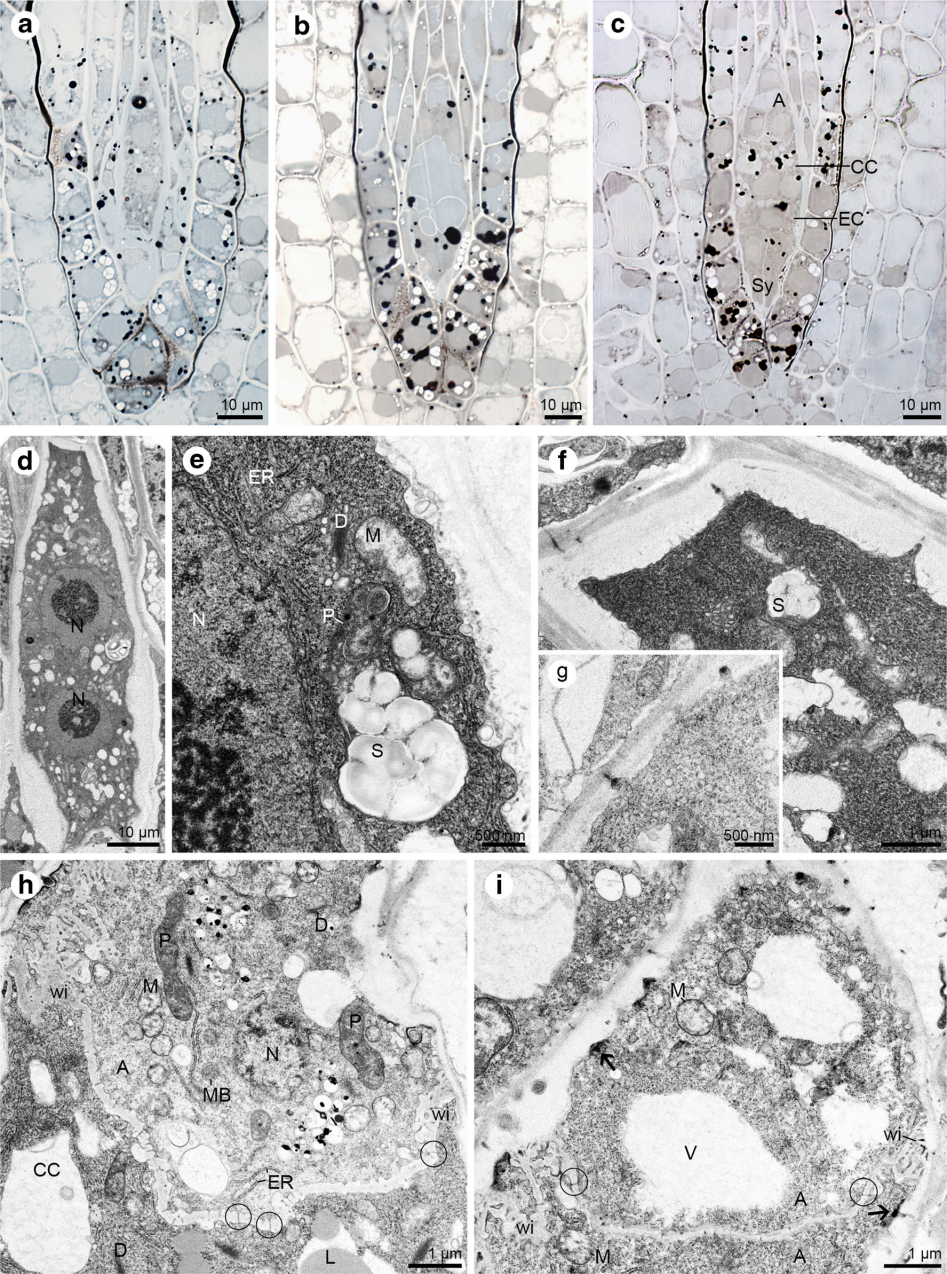
Megagametogenesis—the coenocytic and seven-celled embryo sac of *Sedum sediforme* (Jacq.) Pau. **a**–**c** Light micrographs—results of cytochemical tests; **d**–**i** Electron micrographs. **a** Two-nucleate embryo sac with visible lipid droplets detected with Sudan black B staining. **b** Semi-thin section stained with Sudan black B reveals the presence of lipid droplets within four-nucleate gametophyte. **c** Distribution of the lipids in all cells of seven-celled embryo sac: antipodes (A), central cell (CC) with two polar nuclei, egg cell (EC) and two synergids (Sy). **d** Ultrastructure of two-nucleate embryo sac; Nucleus (N). **e** Higher magnification of two-nucleate gametophyte from (**d**) with fragment of nucleus (N), mitochondria (M), plastids (P) with numerous starch grains (S), profiles of endoplasmic reticulum (ER), active dictyosomes (D). **f** The chalazal part of the two-nucleate embryo sac with present plastids with accumulated starch grains (S) and simple plasmodesmata with electron-dense dome located on the gametophyte cytoplasm side. **g** Fragment of chalazal wall of the four-nucleate embryo sac with visible simple plasmodesmata with an electron-dense dome located on the gametophyte cytoplasm side. **h** Ultrastructure of the antipodes (A) and central cell (CC). In the antipodal cell cytoplasm, microbodies (MB), endoplasmic reticulum (ER), nucleus (N), mitochondria (M), plastids (P), active dictyosomes (D) and wall ingrowths (wi) can be observed. Plasmodesmata occur in walls both between antipodal cells and central cell (circle) and between antipodes and nucellar cells. This second one exhibits the presence of adhering electron-dense material. In the central cell cytoplasm, dictyosomes (D), and lipid droplets (L) are noted. **i** Detail of the antipodes (A) with plasmodesmata with an electron-dense dome (arrow), wall ingrowths (wi) and cytoplasm with mitochondria (M) and some vacuoles (V). Plasmodesmata occur in walls separating the antipodal cells (circle)

Three mitotic divisions without wall formation (cytokinesis) in the functional megaspore led to the formation of the coenocytic embryo sac. Lipid droplets stained with Sudan black B were observed at all stages of megagametogenesis: from the functional megaspore through the coenocytic phase (Fig. 5a–b), until to the cellular embryo sac (Fig. 5c). Analysis of ultrathin sections revealed that in the cytoplasm of a two-nucleate embryo sac plastids occur with numerous large starch granules, active dictyosomes, profiles of the endoplasmic reticulum, and mitochondria (Fig. 5d–e). In addition, at the stage of the two-nucleate embryo sac, the presence of thickened cell walls was found, in which simple plasmodesmata were visible with an electron-dense dome adhering to the gametophyte cytoplasm side (Fig. 5f). Similar structural features were also found in the four-nucleate female gametophyte (Fig. 5g). During megagametogenesis, the embryo sac enlarges and destroys the surrounding cells of the nucellus. The cellular female gametophyte develops after the formation of cell walls within the coenocytic embryo sac. After the cellularisation, the female gametophyte consists of seven cells: two synergids, an egg cell, a central cell and three chalazally located antipodes. Simple plasmodesmata with electron-dense material are present in the external cell walls of antipodal cells (Fig. 5h–i). Antipodes are the only cells of the mature embryo sac that are distinguished by their presence. Furthermore, the outer walls of antipodes and adjacent, small fragments of the transverse walls (separating the antipodes from each other and from central cell) form wall ingrowths. Nucleus, plastids, mitochondria, microbodies, vacuoles and profiles of rough endoplasmic reticulum are visible within the cytoplasm of the antipodes. Antipodes are connected with each other and with the central cell by the simple plasmodesmata (Fig. 5h–i) which ensure the symplasmic continuity of these cells.

**Fig. 6:**
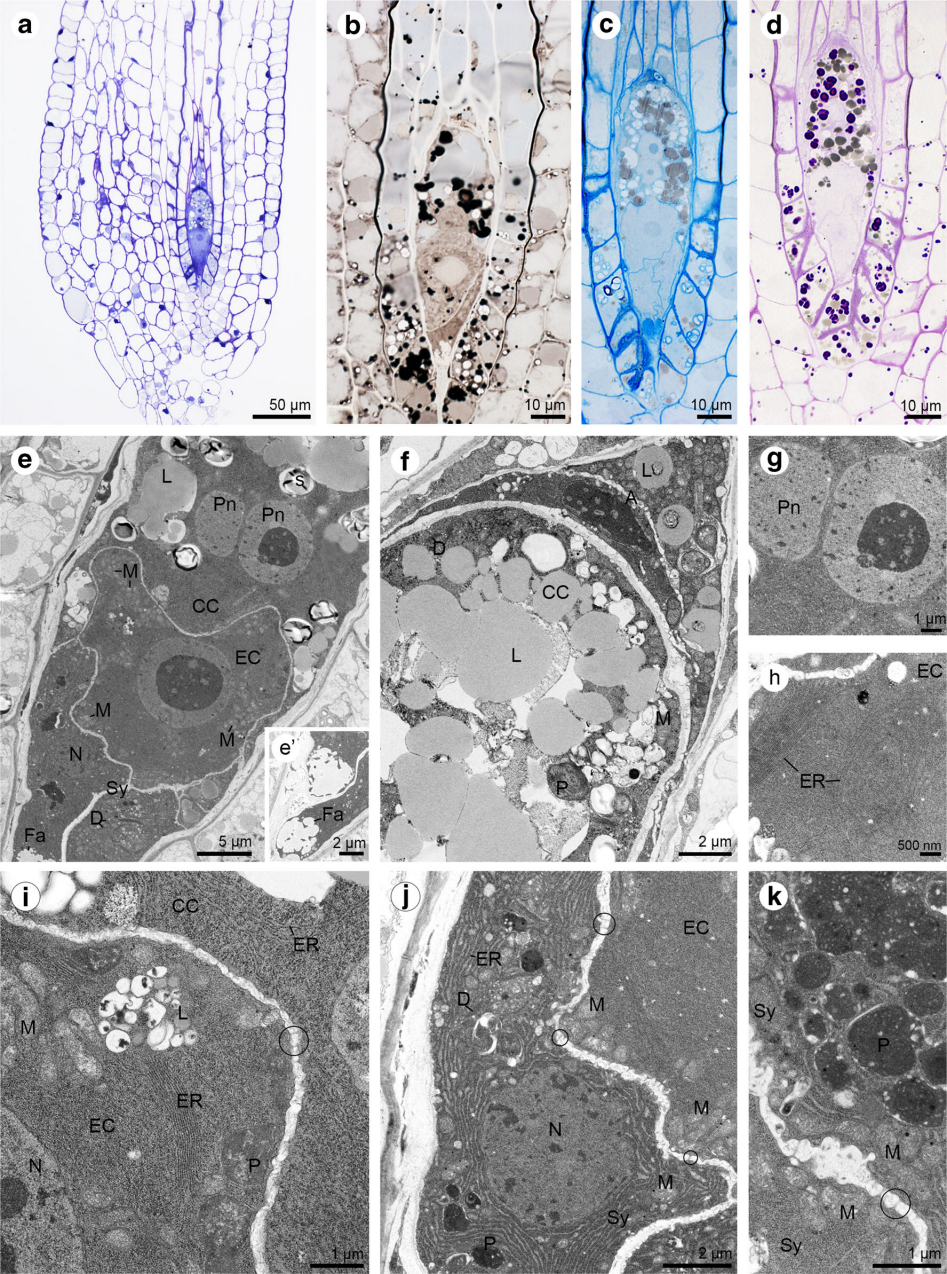
Cellular female gametophyte of *Sedum sediforme (*Jacq.) Pau. **a**–**d** Light micrographs—results of cytochemical tests; **e**–**k** Electron micrographs. **a** Fragment of the longitudinal section of the anatropous ovule with an embryo sac (during antipodes degeneration). **b** Sudan black B staining reveals the presence of lipid droplets in all cells of embryo sac. **c** Semi-thin section treated with aniline blue black. All cells of the embryo sac give a positive reaction for proteins. Filiform apparatus of the synergids is built of ABB-positive ingrowths. **d** Periodic acid-Schiff reaction shows the location of the insoluble polysaccharides in four cells of the female gametophyte. **e** Ultrastructure of the central cell (CC) with two polar nuclei (Pn) and lipid droplets (L); egg cell (EC) with mitochondria (M) located near cell wall; two synergids (Sy) with observed nucleus (N), dictyosomes (D) and fragment of filiform apparatus (Fa). **e’**. The micropylar region of the synergids with visible filiform apparatus (Fa). **f** Chalazal part of the embryo sac with degenerating antipodes (A), central cell (CC) with numerous lipid droplets (L) and plastid (P), mitochondria (M), and dictyosomes (D). **g** Two polar nuclei (PN) of central cell adhere to each other and fuse. **h** Higher magnification of the egg cell (EC) cytoplasm with parallel and concentrically arranged profiles of endoplasmic reticulum (ER). **i** Portion of the egg cell (EC) and central cell (CC) with cytoplasm in which are noted: plastids (P) lipid droplets (L), mitochondria (M), nucleus (N), and profiles of endoplasmic reticulum (ER). Plasmodesmata occur in walls between the egg cell and a central cell (circle). **j** Ultrastructure of active synergids (Sy) with observed plastids (P), mitochondria (M), nucleus (N), profiles of endoplasmic reticulum (ER), and dictyosomes (D). The synergids (Sy) and egg cell (EC) are connected with plasmodesmata (circle). Mitochondria (M) occur near the cell wall of the egg cell. **k** Higher magnification of the synergids (Sy) with numerous plastids (P), and mitochondria (M). Plasmodesmata (circle) are visible in the wall that separates two ultrastructurally similar synergid cells

In the mature embryo sac, the antipodes degenerate so finally the female gametophyte is formed by the egg apparatus and central cell (Fig. 6a). The presence of lipids (Fig. 6b), proteins (Fig. 6c) and insoluble polysaccharides (Fig. 6d) was observed in all cells that form the embryo sac: synergids, an egg cell and a central cell; however, a more significant accumulation was noticed in the central cell. In addition to the numerous lipid droplets in the cytoplasm of the central cell, the plastids, endoplasmic reticulum, mitochondria, and dictyosomes were present (Fig. 6e–f, i). Two polar nuclei of the central cell fuse during embryo sac development (Fig. 6g). The cytoplasm of the egg cell contains two types of endoplasmic reticulum (a concentrically arranged laminae RER structure and a parallel organised one) (Fig. 6h); lipid droplets, plastids (a few of which store starch grains—not shown) (Fig. 6i–j). Some plastids of the egg cell are in close contact with the rough endoplasmic reticulum (Fig. 6i). Mitochondria form larger groups and occur near the cell wall (Fig. 6i–j). The central cell and egg cell are connected to each other by plasmodesmata (Fig. 6i) like the egg cell and synergids (Fig. 6j). Two ultrastructuraly identical synergids occur on the micropylar pole of the embryo sac (Fig. 6j) with formed filiform apparatus (Fig. 6e’). Each of these has one nucleus, active dictyosomes, profiles of ER, mitochondria and plastids which can also form groups (Fig. 6j–k). In addition, the presence of autophagic structures was found in the cytoplasm of synergids (Fig. 6j). Plasmodesmata connect protoplasts of both synergids (Fig. 6k).

**Fig. 7:**
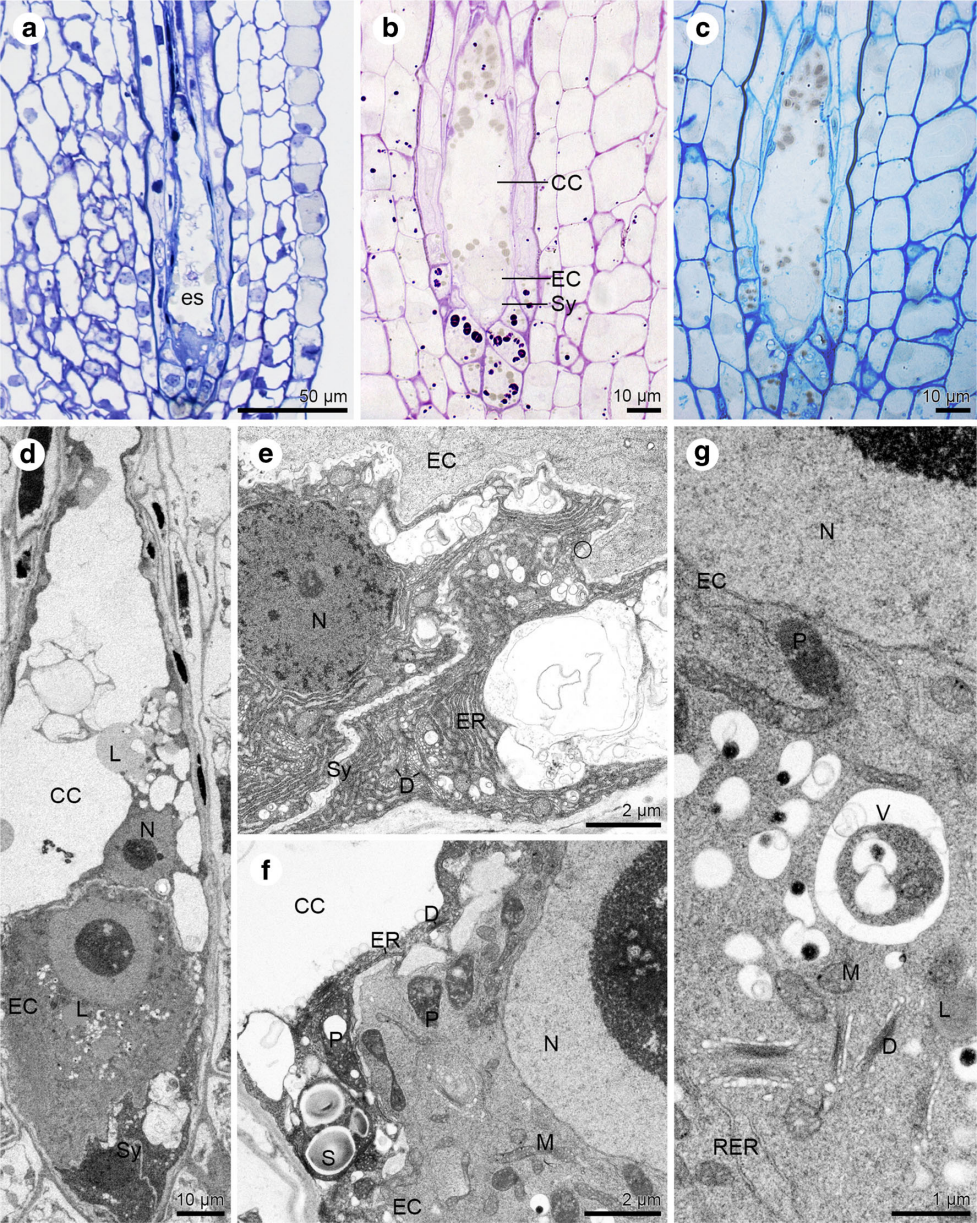
Mature embryo sac of *Sedum sediforme (*Jacq.) Pau. **a**–**c** Light micrographs—results of cytochemical tests; **d**–**g** Electron micrographs. **a** Elongated embryo sac (es) located in the anatropous ovule. **b** Insoluble polysaccharides detected in the central cell (CC), egg cell (EC) and synergids (Sy). **c** Semi-thin section stained with aniline blue black for proteins give a positive reaction. **d** Ultrastructural observations of elongated embryo sac from (**a**) with: two synergids (Sy) where the cytoplasm is similarly electron-dense, egg cell (EC) and vacuolated central cell (CC); lipid droplets (L); nucleus (N). **e** Ultrastructuraly similar and active synergids (Sy) have visibly irregular in thickness cell walls in which occur plasmodesmata (circle). The endoplasmic reticulum (ER) and dictyosomes (D) are noted in cytoplasm of synergids (Sy); Egg cell (EC). **f** The cytoplasm of the central cell (CC) is located near the cell wall. It is more electron-dense compared to the egg cell (EC) cytoplasm. The dictyosomes (D), profiles of endoplasmic reticulum (ER) and plastid (P) with starch grains (S) are present in the central cell (CC). The cell wall separating the central cell (CC) from the egg cell (EC) is not regular in thickness. Plastid (P), mitochondria (M) and nucleus (N) occur in egg cell (EC) cytoplasm. **g** Higher magnification of the egg cell cytoplasm with nucleus (N), plastids (P), vacuoles (V), mitochondria (M), active dictyosomes (D), profiles of rough endoplasmic reticulum (RER) and lipid droplets (L)

The embryo sac elongates during maturation (Fig. 7a). The central cell is clearly vacuolated and has two polar nuclei which fuse and form a secondary nucleus. Insoluble polysaccharides, proteins and lipids are present in the cytoplasm of female gametophyte cells (Fig. 7b–d). Synergids have a well-developed filiform apparatus (not shown), and their cytoplasm is active and contains active dictyosomes, and abundant ER profiles. The cell wall forms an irregular layer, which is discontinuous (Fig. 7e). Also, the wall separating the egg cell from the central cell is interrupted, so that in some places only the cell membrane separates neighbouring cells (Fig. 7f). In the cytoplasm, which is located in close contact with the cell wall of the central cell occur plastids with starch granules, ER profiles, dictyosomes and mitochondria (Fig. 7f). The egg cell is distinguished by the presence of active dictyosomes, mitochondria, plastids (a few of which store starch), vacuoles, lipid droplets and rough ER profiles (Fig. 7f–g).

## Discussion

The embryo sac of *S. sediforme* develops according to the *Polygonum* type in crassinucellate, bitegmic and anatropous ovules. The nucellus is *Sedum* type and its hyponucellus (nucellus under developing gametophyte) is very well developed (Herr 1984). As in *S. sediforme,* the monosporic megasporogenesis and *Polygonum* type of megagametogenesis is described in most representatives of Crassulaceae and genus *Sedum* (Mauritzon 1933; Thiede and Eggli 2007). This means that one megaspore mother cell (MMC) undergoes meiotic division, cytokinesis and forms one, usually linear tetrad of megaspores (megasporogenesis). One megaspore localised chalazally becomes an embryo sac mother cell which after mitosis (three rounds) and ultimately cytokinesis gives rise to the formation of a mature female gametophyte (megagametogenesis) (D’Amato 1977; Raghavan 2000; Chevalier et al. 2011). A linear tetrad of megaspores is not formed in all species of the studied family, because tetrads with other than a linear arrangement of cells or even triads have been observed. In the tested species, the step of cell wall formation was omitted in the micropylar dyad cell after karyokinesis. This results in the formation of a triad composed of two chalazally placed megaspores and one binucleate cell. The triad formation has already been observed in other angiosperms (Bouman 1984; Johri et al. 1992); for example, in *Trifolium alexandrinum* (Krupko 1973), *Cytisus striatus* and *C. multiflorus* (Rodríguez-Riaño et al. 2006), *Polianthes tuberosa* (González-Gutiérrez and Rodríguez-Garay 2016) and also in Crassulaceae. In Crassulaceae, as in other angiosperms, the nucleus of a micropylar dyad cell can undergo meiosis II, as is in *Aeonium arboreum* and *Rosularia sempervivum* (Mauritzon 1933). In *Echeveria rosei* and *Umbilicus intermedius* belonging to Crassulaceae, the micropylar dyad cell is mononucleate. In *Sedum* (*S. annum*, *S. caeruleum*), the formation of the triad with mononucleate or binucleate micropylar dyad cell has been described as only one of the possibilities for cells formed after megasporogenesis (in addition to normal, linear type tetrads) (Mauritzon 1933). In *S. sediforme,* the triad is only one noticed result of megasporogenesis. Tetrad formation has not been observed. The situation of the inhibition of non-functional dyad cell division and development which is localised at micropylar pole in *S. sediforme* can be interpreted as a trend to further reduction (Bouman 1984). The opposite situation is observed when the suppression of the cell wall formation is described in a cell from which the gametophyte originates. Then, it is defined as a trend towards specialisation or bisporic and tetrasporic development (Bouman 1984).

*Sedum sediforme* belongs to the genus *Sedum* section *Rupestria* (Nikulin et al. 2016). So far, the ovules of this species have been studied cytochemically and ultrastructuraly only during the development of the embryo. Previous investigations have described the structure of the suspensor which is formed by a basal cell and maximally 8 chalazal cells. Only in this species from the Crassulaceae has the situation been observed where the nucleus of the basal cell enters the narrowing connecting the basal cell with the micropylar haustorium (Majcher 2017). In addition, ovules of *S. rupestre*, a species which also belongs to the series *Rupestria*, have been examined ultrastructurally and cytochemically during embryogenesis. The suspensor of *S. rupestre* is also filamentous and is built of 5–10 cells (Czaplejewicz and Kozieradzka-Kiszkurno 2013). So far, plasmodesmata with an electron-dense dome have only not been observed in the above mentioned species of Crassulaceae during the development of the embryo. This feature distinguishes *Sedum* species from section *Rupestria* from other Crassulaceae. During the research, it was confirmed that the absence of plasmodesmata with an electron-dense dome during embryogenesis does not preclude their presence in the earlier stages of development or during the female gametophyte development. They have been observed during the coenocytic embryo sac stage and in outer walls of antipodes (during megagametogenesis). During megasporogenesis in *S. sediforme*, simple plasmodesmata occur in the walls of the megaspore mother cell (MMC), the chalazal cell of the dyad and triad and chalazal part of the functional megaspore similarly to *Arabidopsis thaliana* (Bajon et al. 1999), and *Capsella* (Schulz and Jansen 1986). This occurrence provides the opportunity for nutritional substance flow towards the cell providing the origin of the embryo sac mother cell and female gametophyte. Such a situation can favour the survival of chalazal placed cell (Schulz and Jansen 1986; Yang 2006). Plasmodesmata are channels that provide opportunities for symplasmic transport of nutrients and signal molecules required for correct development in plants (Benitez-Alfonso 2014 and literature therein). Plasmodesmata with an electron-dense dome were observed only during megagametogenesis in the examined species. Plasmodesmata with adhering electron-dense material distinguish Crassulaceae from other angiosperms, because they were described only during the development of the female gametophyte (Brzezicka and Kozieradzka-Kiszkurno 2018) and embryo (Kozieradzka-Kiszkurno and Bohdanowicz 2010; Kozieradzka-Kiszkurno et al. 2011; Kozieradzka-Kiszkurno and Płachno 2012) in this family. In *S. hispanicum*, characteristic plasmodesmata have been recorded as early as during megasporogenesis. This might be associated with the different structure, and thickness of the cell walls of megaspores in *Sedum*. Walls of *S. hispanicum* megaspores are not thickened as in megaspores of *S. sediforme*. This hypothesis will be verified during subsequent studies on other Crassulaceae species when more ultrastructural data will be collected.

During the megasporogenesis of *S. sediforme*, the thickened walls were observed from the dyad to the functional megaspore stage. Initially, they probably separated only the cells of dyad and triad from each other, while at the stage of the functional megaspore and also the coenocytic embryo sac (megagametogenesis) they occur in the form of an irregular or interrupted layer which is crossed with plasmodesmata. Thickening of the walls is a result of the presence of electron-translucent material. Based on the available literature, it can be concluded that cell wall material is callose. This interpretation is based on very similar observations made during megasporogenesis in for example *Capsella* (Schulz and Jansen 1986)*, Tillandsia* (Papini et al. 2011), *Epipactis* sp. (Bouman 1984) where the aniline blue fluorescence was observed together with ultrastructural observations. Fluorescence here localised is described as sign of the presence of callose. It is necessary to perform callose staining to precisely describe the location of callose occurrence and its potential function in *Sedum*.

A common feature for both *Sedum* species is the occurrence of characteristic plasmodesmata with adhering electron-dense material during megagametogenesis in the outer walls of the coenocytic embryo sac despite of the different thicknesses of the walls. In other angiosperms such as *Capsella*, the plasmodesmata disappear at the stage of the four-nucleate female gametophyte (Schulz and Jansen 1986). If plasmodesmata remain present at this stage of development they are less frequently (Yang 2006). Temporary regulation of the substance flow is associated with callose depositions as well as the occurrence (lack or presence) of plasmodesmata (Ünal et al. 2013). There is a possibility that plasmodesmata with an electron-dense body are still present in *Sedum* in the coenocytic embryo sac because they regulate the flow of substances, as has been shown and examined experimentally during *S. acre* embryogenesis (Wróbel-Marek et al. 2017). Plasmodesmata with adhering electron-dense material might also regulate the flow of substances during female gametophyte development. This may be the reason that they are still present in the coenocytic embryo sac of *S. sediforme*. This possibility is also supported by the fact of the presence of the thickened wall of the coenocytic embryo sac in *S. sediforme*, which can also contribute to the regulation of the substances flow. Further ultrastructural observations in combination with experimental research are needed to expand knowledge in this area.

At the stage of the megaspore mother cell, dyad, triad, functional megaspore in *S. sediforme*, the intraplastidial space whose structure is similar to that of the surrounding cytoplasm occurs in some plastids. So far, it has been assumed that plastids form a protrusion which swirls around the fragments of the cytoplasm (van Doorn and Papini 2013 and literature therein; Parra-Vega et al. 2015). Moreover, some preliminary evidences have been found for possible degradation of the cytoplasmic material inside the organelle (autophagic plastids). Different states of degradation are supposed on the basis of the changing structure of the contents of intraplastidial spaces. Completely digested cytoplasm has been observed on micrographs as uniform electron-translucent areas. Autophagic activity in plastids has also been confirmed by cytochemical staining conducted on petals cells in *Dendrobium* flowers (van Doorn et al. 2011). In addition, observations of crystalloids made by transmission electron microscopy show some evidence to support the hypothesis that autophagic plastids can degrade other plastids (Papini and van Doorn 2015). Plastids observed during the present study probably engulf portions of the cytoplasm. Our observations on *S*. *sediforme* suggest that the autophagy process occurs but ultrastructural changes to the contents of the intraplastidial space were not noticed. Similar structures of plastids can be observed during megasporogenesis in functional megaspore of *Tillandsia* (Papini et al. 2011), as was indicated in review by van Doorn and Papini (2013). Such function of the plastids (called plastolysomes) has been analysed during studies of suspensor—for example, in *Phaseolus* species (Nagl 1977; Gärtner and Nagl 1980)—and tapetum cells of *Tillandsia albida* (Papini et al. 2014). In members of Crassulaceae, the presence of plastolysomes has not been described previously (Kozieradzka-Kiszkurno and Płachno 2013). However, it is important to note that the role of plastids as an autophagosome or autolysosome is not fully documented (Papini and van Doorn 2015). In *S. sediforme*, together with the plastids which are described above, autophagic vacuoles have been recorded during megasporogenesis (in cytoplasm of MMC, both cells of dyad, functional megaspore and other cells of triad) and megagametogenesis (coenocytic and cellular gametophyte). The portion of the cytoplasm is surrounded and sequestrated, which contributes to autophagic vacuole formation. So far, autophagic vacuoles have been described during the development of the female gametophyte of *Capsella* where the acid phosphatase activity was recorded (Schulz and Jansen 1981, 1986). Autophagic vacuoles can have some function in organelle selection, cytoplasm regeneration (Yang 2006) and cytoplasmic constituent destruction (Schulz and Jansen 1986; van Doorn and Papini 2016).

Female gametophyte development takes place in ovular, sporophytic tissues. The surrounding, maternal cells are not neutral for the developing gametophyte because the sporophytic cells influence it during both megasporogenesis and megagametogenesis (Yang and Sundaresan 2000; Drews and Koltunow 2011; Figueiredo and Köhler 2016). For example, the covering integuments not only physically protect the female gametophyte, but they also have an active signalling function, which is necessary for correct development of gametophytes (Figueiredo and Köhler 2016). Moreover, the surrounding nucellar cell can be consumed, being degraded by developing embryo sac. The products of the nucellar degradation can be the source of materials and energy for further maturation (Bhojwani et al. 2015; Rodríguez-Riaño et al. 2006). The sporophytic tissues also have a nutritional function. The products of non-functional megaspore degeneration may also be used by developing gametophytes (Raghavan 1997). Programmed cell death (PCD) is an important process involved in nucellar and non-functional megaspore/cell degradation (van Hautegem et al. 2015). During development, the *S. sediforme* functional megaspore grows and is surrounded by two non-functional, degenerating cells at the micropylar pole. When the embryo sac mother cell conducts first and second mitotic divisions (without cytokinesis), the bordering nucellar cells are also crushed by the enlarging gametophyte. Products appearing after disintegration of neighbouring cells can be a source of nutrients, which are used for the further development of the female gametophyte. This belief is additionally confirmed by the observation of a larger accumulation of reserve substances (lipid droplets, starch granules) within the functional megaspore and the subsequent binucleate embryo sac of *S. sediforme*.

The ovule of the study species is elongated. The embryo sac of *S. sediforme* is a small part compared to the whole ovule; however, during maturation it also grows towards the chalaza and becomes elongated. There is a suspicion that in some species of Crassulaceae, the embryo sac becomes haustorial, but so far this has not been confirmed (Johri et al. 1992). Moreover, there has been the suggestion of the possibility of starch accumulation by haustorial embryo sac. In *S. sediforme*, the extending embryo sac does not show accumulation of larger amounts of materials (proteins, lipids and insoluble polysaccharides), which has been demonstrated the ABB staining, PAS method and ultrastructural observations. The occurrence of the elongation of mature embryo sacs has not been observed in *S. hispanicum*, the species whose ovules have been analysed cytochemically and ultrastructurally (Brzezicka and Kozieradzka-Kiszkurno 2018). The difference in the embryo sac structure is possibly a result of the systematic position of the species. *S. hispanicum* belongs to the genus *Sedum* and clade *Leucosedum* (Nikulin et al. 2016). The cell wall in the mature *S. sediforme* embryo sac (separating the synergids from the egg cell and the egg cell from the central cell) has a similar structure to that described in *S. hispanicum*, but only at the stage of the elongated embryo sac built of the egg apparatus and a central cell. The cell wall disappears locally and the neighbouring cells are separated by a plasma membrane. This shows that the elongated embryo sac of *S. sediforme* has become fully mature. The thickness and presence of cell walls of egg apparatus change during maturation. In angiosperms, the chalazal part of the egg cell wall thins or disappears locally or in the whole chalazal part (Jensen 1973; Willemse and van Went 1984; Raghavan 1997; Johri et al. 2001). A similar change in cell wall structure is observed in the chalazal end of synergids, where regions with irregular, visibly thin or absent walls also occur. This could be to a facilitate transport of materials (nutrients) and entry of sperm (Willemse and van Went 1984; Raghavan 1997).

The mature female gametophyte of *Polygonum* type is built of seven cells, but this typically observed structure can be modified by cell proliferation or cell death (Yadegari and Drews 2004). Two synergids, an egg cell, a central cell and three antipodes are noticed in the embryo sac of the study species. The egg cell and central cell are two reproductive cells that participate in fertilisation. Synergids and antipodes are accessory cells, but only the former play an essential role in fertilisation, for example pollen tube guidance and reception (Sánchez-León and Vielle-Calzada 2010). Synergids and antipodes are two lineages of cells, which undergo PCD what is important for reproductive success (van Hautegem et al. 2015). Antipodes are as called an antipodal apparatus. It is a highly variable element of the embryo sac (Willemse and van Went 1984; Johri et al. 1992, 2001). Their function is still not precisely specified, but several possible functions (in nutrition, secretion and storage) of these cells have been proposed based mainly on morphological analysis (Willemse and van Went 1984; Vijayaraghavan et al. 1988; Raghavan 1997; Bhojwani et al. 2015). Moreover, depending on the species, the antipodes can be ephemeral (many of dicotyledons species) or persistent (Willemse and van Went 1984; Tang et al. 2009). In addition, the situation of antipodal proliferation occurs in monocotyledon plants, for example Poaceae (Chettoor and Evans 2015). In *S. sediforme*, the antipodes are ephemeral, similar to the situation described in *S. hispanicum*, but their ultrastructure differs. Antipodes in the study species are metabolically active cells which can be interpreted after cytoplasm observations where organelles such as plastids, ribosome, active dictyosomes, and ER profiles occur. Wall ingrowths are visible in outer walls of antipodes, but they are more conspicuous in lateral walls than in the chalazal wall region. Interantipodal cell walls also show the presence of wall ingrowth formation. Moreover, next to wall ingrowths, plasmodesmata are present in outer walls of antipodes, which separate these embryo sac cells from the nucellus. Observations made during this study indicate the nutritional function of antipodes. Due to the presence of the above described structures (plasmodesmata, wall ingrowths), it can be concluded that symplasmic transport and intensive apoplasmic transport takes place. A new feature observed on electronograms in the form of simple plasmodesmata with electron-dense dome suggests that this transport can be precisely regulated. Data collected during our research shows the possibility that this transport is regulated in a similarly manner to transport of substances through the suspensor basal cell walls (Wróbel-Marek et al. 2017). In addition, plasmodesmata occurring between antipodes and antipodal cells-central cell confirm the presence of a pathway for metabolites which can run in two directions: nucellus-antipodes-central cell and vice versa (Willemse and van Went 1984). The presence of wall ingrowths has also been pointed out in other angiosperms species (Vijayaraghavan et al. 1988), for example in *Aconitum vulparia* (Bohdanowicz and Turała-Szybowska 1985, 1987), *Oryza sativa* (Maeda and Miyake 1996), and *Eleusine tristachya* (Lovisolo and Galati 2007). This shows that these cells have transfer character. In *S. sediforme*, the wall ingrowths are branched and in all antipodal cell walls the plasmodesmata are present. A similar situation has been observed in persistent antipodes of *A. vulparia*; however, the plasmodesmata did not occur in chalazal wall connecting the antipodal cells with nucellar cells. The lifespan of antipodes is controlled by genes expressed in neighbouring cells of the gametophyte (Tekleyohans et al. 2017).

## Conclusions

The mature female gametophyte of *Sedum sediforme* is elongated and after antipodes degeneration is formed by an egg cell, two synergids and a central cell. Embryo sac development is monosporic *Polygonum* type. A triad of cells is always formed during megasporogenesis. The embryo sac mother cell is placed chalazally. The functional megaspore forms firstly a coenocytic and later cellular gametophyte after cell wall formation. Plasmodesmata with an electron-dense dome were observed in outer cell walls during both coenocytic and cellular embryo sac stages. The three antipodal cells have a unique structure in study species, which has not been described in other angiosperm plants. During ultrastructural observations of antipodals, plasmodesmata with an electron-dense dome were observed. The presence of these structures in connection with the occurrence of wall ingrowths shows that antipodal cells may perform a role in transport. Plasmodesmata and wall ingrowths provide the opportunity for intense symplasmic and apoplasmic transport. Electron-dense material may perform a role in the regulation of substances transport. On the basis of the available literature, it can be assumed that transport through plasmodesmata with an electron-dense dome takes place in one direction, but this requires further experimental analysis with the use of microinjections of fluorescent tracers. During the present study, new structural features of antipodal cells were described, which could be useful in creating a better understanding of the function of these cells. Moreover, this first ultrastructural comparative analysis of ovules at the stage of gametophyte development in *Sedum* shows that these species differs in the manner of female gametophyte development and structure.

## References

[CR1] Bajon C, Horlow C, Motamayor JC, Sauvanet A, Robert D (1999). Megasporogenesis in *Arabidopsis thaliana* L.: an ultrastructural study. Sex Plant Reprod.

[CR2] Benitez-Alfonso Y (2014). Symplastic intercellular transport from a developmental perspective. J Exp Biol.

[CR3] Bhojwani SS, Bhatnagar SP, Dantu PK (2015). Female gametophyte in: the embryology of angiosperms.

[CR4] Bohdanowicz J, Turała-Szybowska K (1985). Ultrastructure of endopolyploid antipodals in *Aconitum vulparia* Rchb. I Antipodals in the mature embryo sac. Protoplasma.

[CR5] Bohdanowicz J, Turała-Szybowska K (1987). Ultrastructure of endopolyploid antipodals in *Aconitum vulparia* Rchb. II Antipodals in the period of free nuclear endosperm. Protoplasma.

[CR6] Bouman F, Johri BM (1984). The ovule. Embryology of angiosperms.

[CR7] Bronner R (1975). Simultaneous demonstration of lipid and starch in plant tissues. Stain Technol.

[CR8] Brzezicka E, Kozieradzka-Kiszkurno M (2018). Ultrastructural and cytochemical aspects of female gametophyte development in *Sedum hispanicum* L. (Crassulaceae). Protoplasma.

[CR9] Chettoor AM, Evans MMS (2015). Correlation between a loss of auxin signaling and a loss of proliferation in maize antipodal cells. Front Plant Sci.

[CR10] Chevalier É, Loubert-Hudon A, Zimmerman EL, Matton DP (2011). Cell–cell communication and signalling pathways within the ovule: from its inception to fertilization. New Phytol.

[CR11] Christenhusz MJM, Byng JW (2016). The number of known plants species in the world and its annual increase. Phytotaxa.

[CR12] Czaplejewicz D, Kozieradzka-Kiszkurno M (2013). Ultrastructural and cytochemical studies of the embryo suspensor of *Sedum reflexum* L. (Crassulaceae). Acta Biol Cracov Ser Bot.

[CR13] D’Amato F (1977). Nuclear cytology in relation to development.

[CR14] Drews GN, Koltunow AMG (2011). The female gametophyte. ASPB.

[CR15] Figueiredo DD, Köhler C (2016). Bridging the generation gap: communication between maternal sporophyte, female gametophyte and fertilization products. Curr Opin Plant Biol.

[CR16] Gallo L, Zika P (2014). A taxonomic study of *Sedum* series *Rupestria* (Crassulaceae) naturalized in North America. Phytotaxa.

[CR17] Gärtner PJ, Nagl W (1980). Acid phosphatase activity in plastids (plastolysomes) of senescing embryo-suspensor cells. Planta.

[CR18] González-Gutiérrez AG, Rodríguez-Garay B (2016). Embryogenesis in *Polianthes tuberosa* L var. simple: from megasporogenesis to early embryo development. SpringerPlus.

[CR19] González-Gutiérrez AG, Gutiérrez-Mora A, Rodríguez-Garay B (2014). Embryo sac formation and early embryo development in *Agave tequilana* (Asparagaceae). SpringerPlus.

[CR20] Hart H’t, Bleij B, Eggli U (2003). Sedum. Illustrated handbook of succulent plants. *Crassulaceae*.

[CR21] Herr JM (1984). Embryology and taxonomy in: Johri BM (ed) Embryology of angiosperms.

[CR22] Jensen WA (1962). Botanical histochemistry.

[CR23] Jensen WA (1973). Fertilization in flowering plants. Bioscience.

[CR24] Johri BM, Ambegaokar KB, Srivastava PS (1992). Comparative embryology of angiosperms.

[CR25] Johri BM, Srivastava PS, Singh N, Johri BM, Srivastava PS (2001). Reproductive biology of angiosperms. Reproductive biology of plants.

[CR26] Kozieradzka-Kiszkurno M, Bohdanowicz J (2006). Development and cytochemistry of the embryo suspensor in *Sedum*. Acta Biol Cracov Ser Bot.

[CR27] Kozieradzka-Kiszkurno M, Bohdanowicz J (2010). Unusual electron-dense dome associates with compound plasmodesmata in the embryo-suspensor of genus *Sedum* (Crassulaceae). Protoplasma.

[CR28] Kozieradzka-Kiszkurno M, Płachno BJ (2012). Are the symplastic connections between the endosperm and embryo in same angiosperms?-a lesson from the Crassulaceae family. Protoplasma.

[CR29] Kozieradzka-Kiszkurno M, Płachno BJ (2013). Diversity of plastid morphology and structure along the micropyle–chalaza axis of different Crassulaceae. Flora.

[CR30] Kozieradzka-Kiszkurno M, Płachno BJ, Bohdanowicz J (2011). Are unusual plasmodesmata in the embryo-suspensor restricted to species from the genus *Sedum* among Crassulaceae?. Flora.

[CR31] Kozieradzka-Kiszkurno M, Płachno BJ, Bohdanowicz J (2012). New data about the suspensor of succulent angiosperms: ultrastructure and cytochemical study of the embryo-suspensor of *Sempervivum arachnoideum* L. and *Jovibarba sobolifera* (Sims) Opiz. Protoplasma.

[CR32] Krupko S (1973). Megasporogenesis and development of the embryo sac in the Palestine variety of *Trifolium alexandrinum* L. Acta Soc Bot Pol.

[CR33] Lovisolo MR, Galati BG (2007). Ultrastructure and development of the megagametophyte in *Eleusine tristachya* (lam.) lam. (Poaceae). Flora.

[CR34] Maeda E, Miyake H (1996). **U**ltrastructure of antipodal cells of rice **(***Oryza sativa*) after anthesis, as related to nutrient transport in embryo sac. Jpn J Crop Sci.

[CR35] Maheshwari P (1937). A critical review of the types of embryo sacs in angiosperms. New Phytol.

[CR36] Majcher D (2017) Zróżnicowanie budowy wieszadełka zarodkowego w obrębie rodzaju *Sedum* (Crassulaceae). Dissertation, University of Gdańsk, Gdańsk (in Polish)

[CR37] Mauritzon J (1933) Studien über die Embryologie der Familien Crassulaceae und Saxifragaceae. Thesis, University of Lund, Lund

[CR38] Nagl W (1977). ‘Plastolysomes’ – plastids involved in the autolysis of the embryo-suspensor in *Phaseolus*. Z Pflanzenphysiol.

[CR39] Nikulin VY, Gontcharova SB, Stephenson R, Gontcharov AA (2016). Phylogenetic relationships between *Sedum* L. and related genera (Crassulaceae) based on ITS rDNA sequence comparisons. Flora.

[CR40] Papini A, van Doorn W (2015). Crystalloids in apparent autophagic plastids: remnants of plastids or peroxisomes?. J Plant Physiol.

[CR41] Papini A, Mosti S, Milocani E, Tani G, Di Falco P, Brighigna L (2011). Megasporogenesis and programmed cell death in *Tillandsia* (Bromeliaceae). Protoplasma.

[CR42] Papini A, Mosti S, van Doorn WG (2014). Classical macroautophagy in *Lobivia rauschii* (Cactaceae) and possible plastidial autophagy in *Tillandsia albida* (Bromeliaceae) tapetum cells. Protoplasma.

[CR43] Parra-Vega V, Corral-Martínez P, Rivas-Sendra A, Seguí-Simarro JM (2015). Formation and excretion of autophagic plastids (plastolysomes) in *Brassica napus* embryogenic microspores. Front Plant Sci.

[CR44] Raghavan V (1997). Megasporogenesis and megagametogenesis. Molecular embryology of flowering plants.

[CR45] Raghavan V (2000). Megasporogenesis and formation of the embryo sac in: developmental biology of flowering plants.

[CR46] Raghavan V (2006). Double fertilization: embryo and endosperm development in flowering plants.

[CR47] Reiser L, Fischer RL (1993). The ovule and the embryo sac. Plant Cell.

[CR48] Rodríguez-Riaño T, Valtueña FJ, Ortega-Olivencia A (2006). Megasporogenesis, megagametogenesis and ontogeny of the aril in *Cytisus striatus* and *C. multiflorus* (Leguminosae: Papilionoideae). Ann Bot.

[CR49] Rojek J, Kapusta M, Kozieradzka-Kiszkurno M, Majcher D, Górniak M, Sliwinska E, Sharbel TF, Bohdanowicz J (2018). Establishing the cell biology of apomictic reproduction in diploid *Boechera stricta* (Brassicaceae). Ann Bot.

[CR50] Sánchez-León N, Vielle-Calzada JP (2010). Development and function of the female gametophyte. Plant developmental biology-biotechnological perspectives.

[CR51] Schmidt A, Schmid MW, Grossniklaus U (2015). Plant germline formation: common concepts and developmental flexibility in sexual and asexual reproduction. Development.

[CR52] Schulz P, Jansen WA (1981). Pre-fertilization ovule development in *Capsella*: ultrastructure and ultracytochemical localization of acid phosphatase in the meiocyte. Protoplasma.

[CR53] Schulz P, Jansen WA (1986). Prefertilization ovule development in *Capsella*: the dyad, tetrad, developing megaspore, and two-nucleate gametophyte. Can J Bot.

[CR54] Sharp LW (1913). Embryo sac of Crassulaceae. Bot Gaz.

[CR55] Souéges R (1927). Développement de l’embryon chez le *Sedum acre* L. Bull Soc Bot France.

[CR56] Spurr AR (1969). A low-viscosity epoxy resin embedding medium for electron microscopy. J Ultrastruct Res.

[CR57] Tang Y, Gao H, Xie JS (2009). An embryological study of *Eriolaena candollei* Wallich (Malvaceae) and its systematic implications. Flora.

[CR58] Tekleyohans DG, Nakel T, Groß-Hardt R (2017). Patterning the female gametophyte of flowering plants. Plant Physiol.

[CR59] Thiede J, Eggli U, Kubitzki K (2007). Crassulaceae. The familie and genera of vascular plants.

[CR60] Ünal M, Vardar F, Aytürk Ö (2013) Callose in plant sexual reproduction in: current progress in biological research. InTech pp 319–343

[CR61] van Doorn WG, Papini A (2013). Ultrastructure of autophagy in plant cells: a review. Autophagy.

[CR62] van Doorn WG, Papini A (2016). Plastid degeneration in *Tillandsia albida* (Bromeliaceae) and *Lobivia rauschii* (Cactaceae) provides evidence abort the origin and destiny of multilamellar bodies in plants. Phytomorphology.

[CR63] van Doorn WG, Kirasak K, Sonong A, Srihiran Y, van Lent J, Ketsa S (2011). Do plastids in *Dendrobium* cv. Lucky Duan petals function similar to autophagosomes and autolysosomes?. Autophagy.

[CR64] van Hautegem T, Waters AJ, Goodrich J, Nowack MK (2015). Only in dying, life: programmed cell death during plant development. Trends Plant Sci.

[CR65] Vijayaraghavan MR, Seth N, Jain A (1988) Ultrastructure and histochemistry of angiosperm embryo sac - an overview. Proc Natl Acad Sci India Sect B Biol Sci 54(1):93–110

[CR66] Wang D, Tyson MD, Jackson SS, Yadegari R (2006). Partially redundant functions of two SET-domain polycomb-group proteins in controlling initiation of seed development in *Arabidopsis*. Proc Natl Acad Sci U S A.

[CR67] Wang Y, Tsukamoto T, Noble JA, Liu X, Mosher R, Palanivelu R (2017). *Arabidopsis* LORELEI, a maternally-expressed imprinted gene, promotes early seed development. Plant Physiol.

[CR68] Willemse MTM, van Went JL, Johri BM (1984). The female gametophyte. Embryology of angiosperms.

[CR69] Wojciechowicz MK, Samardakiewicz M (1998). The development of female gametophyte and antipodal embryo formation in *Sedum fabaria*. Biol Plant.

[CR70] Wróbel-Marek J, Kurczyńska E, Płachno BJ, Kozieradzka-Kiszkurno M (2017). Identification of symplasmic domains in the embryo and seed of *Sedum acre* L.(Crassulaceae). Planta.

[CR71] Yadegari R, Drews GN (2004). Female gametophyte development. Plant Cell.

[CR72] Yang WC, Basra AS (2006). Female gametophyte development. Handbook of seed science and technology.

[CR73] Yang WC, Sundaresan V (2000). Genetics of gametophyte biogenesis in *Arabidopsis*. Curr Opin Plant Biol.

